# miR-215 suppresses papillary thyroid cancer proliferation, migration, and invasion through the AKT/GSK-3β/Snail signaling by targeting *ARFGEF1*

**DOI:** 10.1038/s41419-019-1444-1

**Published:** 2019-02-27

**Authors:** Jihua Han, Meiyin Zhang, Chunlei Nie, Jinliang Jia, Fengyue Wang, Jiawei Yu, Wen Bi, Bo Liu, Ruinan Sheng, Guoqing He, Lingyu Kong, Lingling Zheng, Rui Pang, Zhaoming Ding, Lili Chen, Qiang Guan, Shangha Pan, Xianzhi Meng, Jin Xu, Lianxin Liu, Jiewu Zhang

**Affiliations:** 1 Department of Head and Neck Surgery, the Third Affiliated Hospital of Harbin Medical University, Harbin, Heilongjiang China; 2 Department of Gynecology, Harbin Medical University Cancer Hospital, Harbin, China; 3 Key Laboratory of Hepatosplenic Surgery, Ministry of Education, Department of General Surgery, the First Affiliated Hospital of Harbin Medical University, Harbin, Heilongjiang China; 4 Department of Cell Biology, Harbin Medical University, Harbin, China; 5 Department of Hepatobiliary Surgery, the First Affiliated Hospital of USTC, Division of Life Sciences and Medicine, University of Science and Technology of China, Hefei, Anhui China

## Abstract

The incidence of papillary thyroid cancer (PTC) has been rapidly increasing in recent years. PTC is prone to lymph node metastasization, which further increases the recurrence rate and mortality of thyroid cancer. However, the underlying mechanisms of this process remain elusive. Several reports have shown that the microRNA miR-215 plays an important role in cancer metastasis. Here, we investigated, for the first time, the potential association between miR-215 and metastasis in PTC. The results of qPCR analysis demonstrated that miR-215 was downregulated in PTC cell lines and tissues, and lower levels of miR-215 correlated with lymph node metastasis of PTC. In vitro and in vivo assays revealed that restoration of miR-215 dramatically inhibited PTC cell proliferation and metastasis. We identified ADP ribosylation factor guanine nucleotide-exchange factor 1 (ARFGEF1) as the target, which mediated the function of miR-215. The expression of ARFGEF1 was inhibited by miR-215, and the effects of miR-215 were abrogated by re-expression of ARFGEF1. Moreover, we found that miR-215 suppressed PTC metastasis by modulating the epithelial–mesenchymal transition via the AKT/GSK-3β/Snail signaling. In summary, our study proves that miR-215 inhibits PTC proliferation and metastasis by targeting ARFGEF1 and indicates miR-215 as a biomarker for PTC prognosis.

## Introduction

Thyroid cancer (TC), deriving from thyroid follicular epithelial cells or parafollicular C cells, is the most frequent malignant tumor in the endocrine system. Papillary thyroid cancer (PTC) is the most common type of TC and, in recent years, its incidence has been increasing worldwide^1^. For most of the patients, the prognosis of PTC is good; however, ~30% of the patients are diagnosed with lymph node metastases (LNM)^2^, which increase the recurrence rate and mortality of PTC^3^. The knowledge of the underlying mechanisms in PTC LNM is essential to make appropriate therapeutic decisions and improve the prognosis of patients with PTC.

MicroRNAs (miRNAs) are short (~22 nucleotides), single-stranded RNAs that regulate gene expression at the post-transcriptional level by binding to the 3′-untranslated region (3′-UTR) of target mRNAs, leading to their degradation or inhibition of their translation^4^. Increasing evidence suggests that miRNAs are involved in various biological processes, including cell proliferation, migration, invasion, differentiation, and immune responses^5^. miRNAs can act as oncogenes or tumor-suppressor genes in PTC^6^. Studies have shown that miR-215 plays a critical role as a tumor suppressor in renal cell carcinoma, gastric cancer, glioma, and colorectal cancer and is a prognostic biomarker for these pathologies^7–10^. However, the potential effect of miR-215 in PTC metastasization has not been investigated yet.

In this study, we investigated the potential function of miR-215 in the progression and development of PTC cancer tissues, showed the downregulation of miR-215 in PTC samples, and the relationship between its aberrant expression and metastasis of PTC. Moreover, we demonstrated, in vitro and in vivo, that overexpression of miR-215 significantly suppresses tumor proliferation and metastasis of PTC by targeting the ADP ribosylation factor guanine nucleotide-exchange factor 1 (ARFGEF1). More interestingly, we also found that miR-215 can modulate the epithelial–mesenchymal transition (EMT) process through the AKT/GSK-3β/Snail signaling.

## Results

### miR-215 is downregulated in PTC tissues and cell lines

**Fig. 1:**
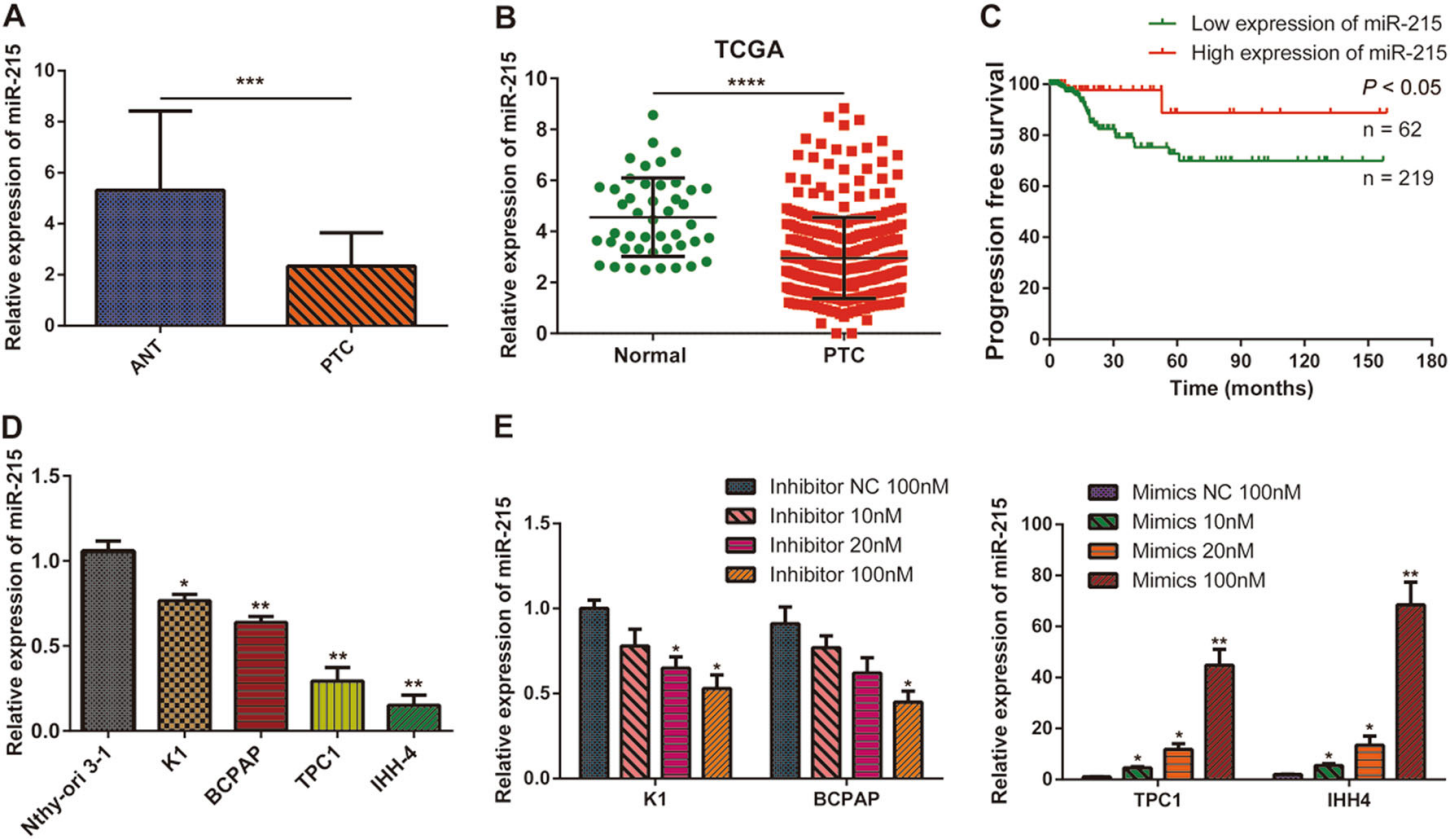
miR-215 expression is decreased in PTC. **a** Relative miR-215 expression in PTC tissues and their corresponding ANT were detected by qPCR (*n* = 48). **b** Relative expression of miR-215 in PTC tissues and normal thyroid tissues in the TCGA database. **c** Kaplan–Meier analysis of PFS in PTC patients with variable expression of miR-215. **d** qPCR analysis of miR-215 expression in a normal thyroid epithelial cell line (Nthy-ori 3–1), and in four PTC cell lines (K1, BCPAP, TPC-1, and IHH4). **e** qPCR analysis of miR-215 expression after its silencing or overexpression in PTC cells. All experiments were performed in triplicate, and the results are presented as the mean ± SD. *P < 0.05, **P < 0.01, ***P < 0.001, and ****P < 0.0001

To investigate the role of miR-215 in PTC, we performed qPCR assays and measured miR-215 expression in 48 paired PTC tissues and the corresponding adjacent normal tissues (ANT). We found that miR-215 expression was significantly lower in PTC tissues than in ANT (Fig. 1a). Similarly, data from The Cancer Genome Atlas (TCGA, https://cancergenome.nih.gov/) database confirmed that miR-215 expression is downregulated in PTC tissues (Fig. 1b). Meanwhile, the survival data from the TCGA database indicated that patients with lower miR-215 expression exhibited significantly poorer disease-free survival (DFS) than patients with higher miR-215 expression (Fig. 1c). Furthermore, the downregulation of miR-215 expression was negatively associated with tumor size (*P* < 0.05), differentiation (*P* < 0.05), and lymph node metastasis status (*P* < 0.01) in PTC (Supplementary Table 1). Moreover, the expression levels of miR-215 were relatively lower in four PTC cell lines (K1, BCPAP, TPC-1, and IHH4) than in the normal thyroid epithelial cell line Nthy-ori 3–1 (Fig. 1d).

Next, we silenced and overexpressed miR-215 by oligonucleotide transfection in cells, which displayed high (K1 and BCPAP) and low (TPC-1 and IHH4) miR-215 endogenous expression, respectively. qPCR analysis revealed that miR-215 was efficiently repressed or overexpressed with 100 nM of inhibitor or mimic oligonucleotides in these cells, which were used for subsequent experiments (Fig. 1e).

### miR-215 suppresses PTC growth both in vitro and in vivo

**Fig. 2:**
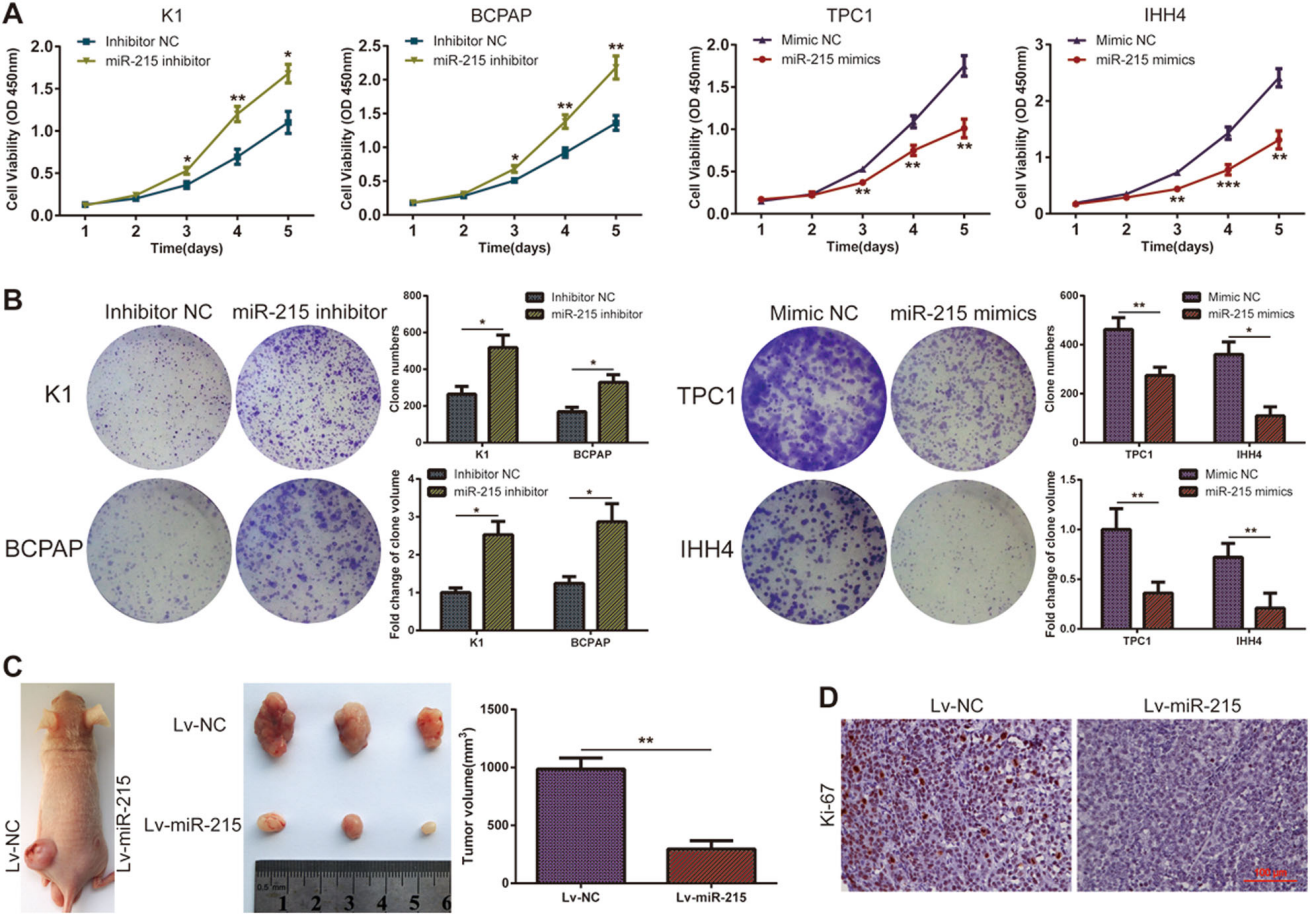
miR-215 inhibits PTC proliferation and tumorigenesis both in vitro and in vivo. **a** Growth curves obtained in proliferation (CCK-8) assays in the indicated PTC cells. **b** Representative images (left) and statistical analysis (right) of colony formation from the indicated PTC cells. **c** Representative images and tumor volume of subcutaneous xenografts. **d** Representative micrographs of Ki-67 expression in the tumor sections, as measured by IHC staining (×100). All experiments were performed in triplicate, and the results are presented as the mean ± SD. *P < 0.05, **P < 0.01, and ***P < 0.001

To detect the role of miR-215 in PTC cell proliferation, we carried out CCK-8 and colony-formation assays. The reduction of miR-215 expression significantly promoted cell growth in K1 and BCPAP cells, and the overexpression of miR-215 dramatically suppressed the growth of TPC-1 and IHH4 cells (Fig. 2a, b and Supplementary Figure 1).

Next, we established a xenograft model by subcutaneously injecting cells stably overexpressing miR-215 (TPC-1-Lv-miR-215) or control cells (TPC-1-Lv-NC) into the flanks of nude mice. The tumor volumes were evidently smaller in the Lv-miR-215 group than in the control group (Fig. 2c). Furthermore, IHC assays showed that the expression of Ki-67 in the Lv-miR-215 group was lower than that in the Lv-NC group (Fig. 2d). Hence, we concluded that miR-215 inhibits PTC tumor growth both in vitro and in vivo.

### Ectopic expression of miR-215 inhibits PTC cell invasion in vitro and metastasis in vivo

**Fig. 3:**
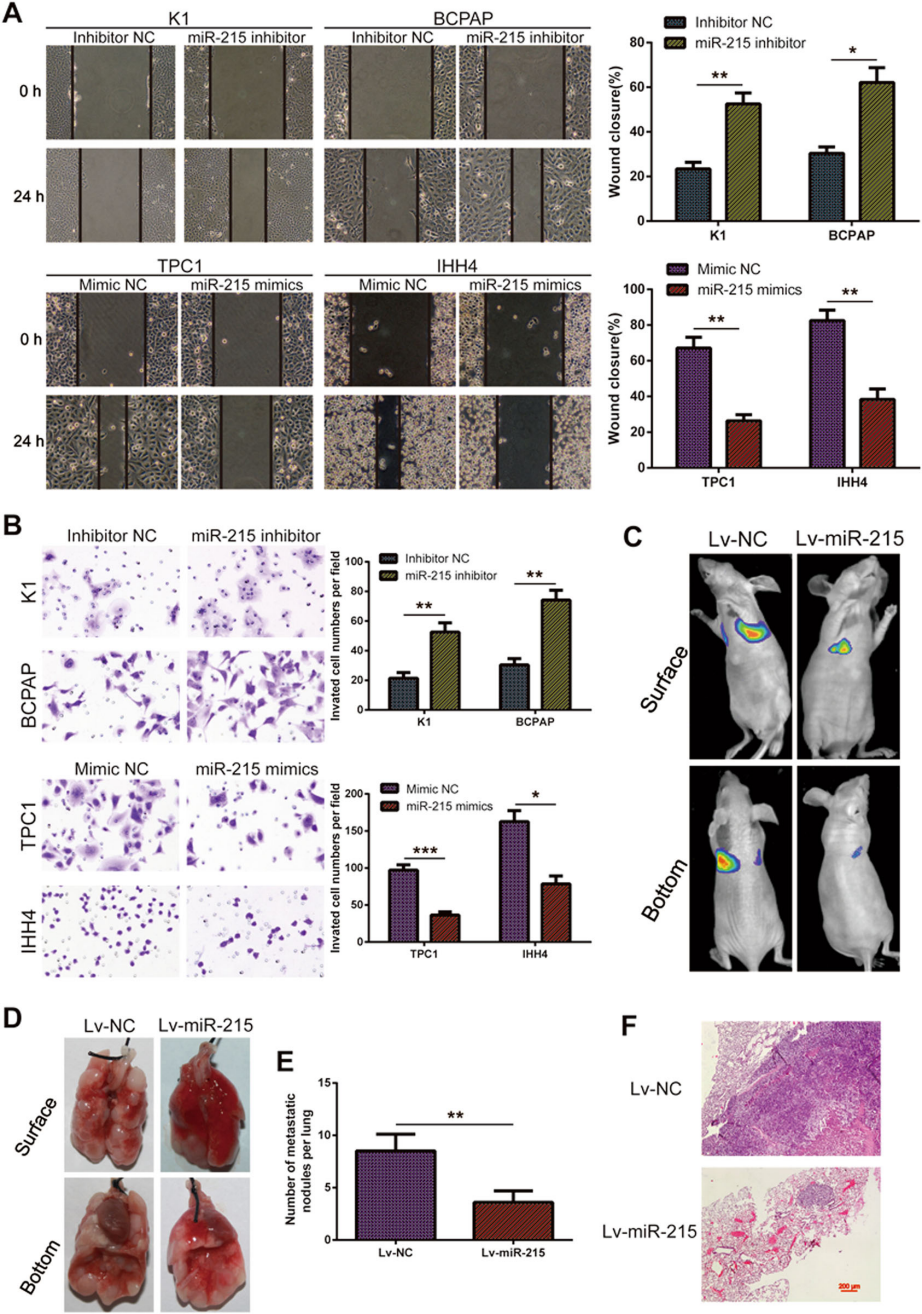
miR-215 represses invasion and metastasis of PTC both in vitro and in vivo. **a** The migration of PTC cells, treated as indicated, was analyzed in wound-healing assays. **b** The invasive capacities of PTC cells, treated as indicated, were detected by transwell invasion assays. **c** Representative bioluminescence images of the lung metastasis model. **d** Representative photographs of dissected lungs from the lung metastasis model. **e** Number of lung metastatic nodules in the indicated groups (*n* = 6 per group). **f** Representative H&E staining of lung metastasis samples (×40). All experiments were performed in triplicate, and the results are presented as the mean ± SD. *P < 0.05, **P < 0.01, and ***P < 0.001

As mentioned above, miR-215 is related to the lymph node metastasis of PTC. Therefore, we examined the effect of miR-215 on the motility and invasiveness of PTC. Wound-healing assays indicated that miR-215 knockdown enhanced the migration of PTC cells, while miR-215 overexpression suppressed cell motility compared with the control cells (Fig. 3a). Transwell migration and Matrigel invasion assays showed that the migratory and invasive capabilities of K1 and BCPAP cells significantly increased after miR-215 downregulation. Meanwhile, upregulation of miR-215 in TPC-1 and IHH4 cells markedly decreased their migratory and invasive behaviors (Fig. 3b and Supplementary Figure 2).

We also established a metastasis mice model by injecting stably overexpressing miR-215 (TPC-1-Lv-miR-215) or control cells (TPC-1-Lv-NC) into the mice tail vein and monitoring the appearance of lung metastatic nodules. We found that less and smaller lung metastatic tumor nodules were detected in the mice injected with the miR-215 overexpressing cells compared with controls (Fig. 3c–e). Histologic analysis of the lungs isolated from the mice further confirmed the effect of miR-215 on lung metastasis (Fig. 3f). These results proved that miR-215 is an important factor for the suppression of PTC invasion and metastasis.

### ARFGEF1 is a direct target of miR-215

To identify the direct downstream target through which miR-215 regulates PTC progression, we conducted bioinformatics analyses based on publicly available databases, including TargetScan (http://www.targetscan.org/vert_72/), miRanda (http://www.microrna.org/microrna/home.do), and miRDB (http://www.mirdb.org/miRDB/). We identified several potential candidate genes, and after reviewing of the literature, decided to focus on ARFGEF1 for further investigation.

**Fig. 4:**
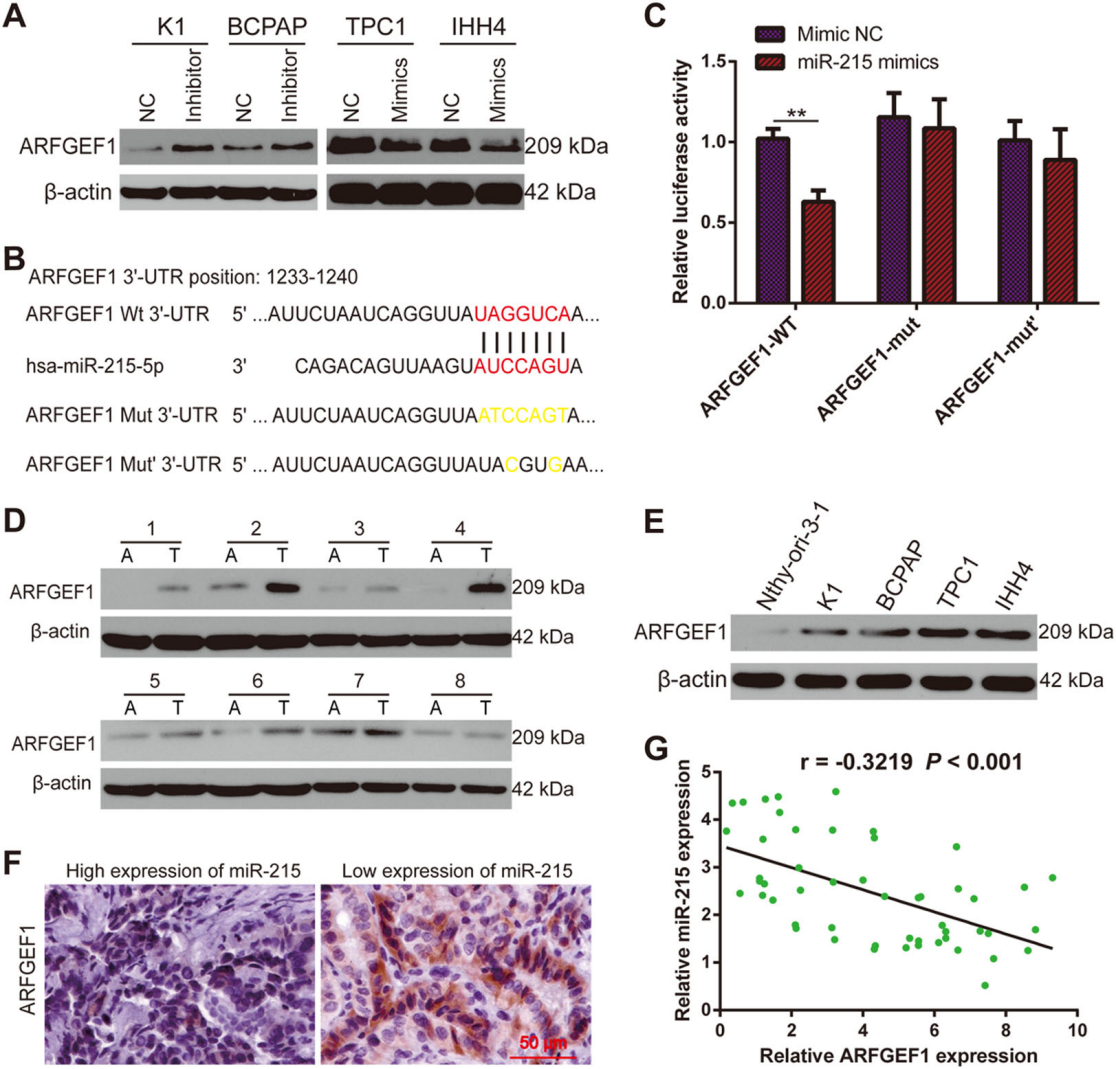
ARFGEF1 is a direct downstream target of miR-215. **a** Western blotting analysis showing the expression of ARFGEF1 after miR-215 silencing or overexpression. **b** Schematic representation of miR-215 binding site in *ARFGEF1* and the corresponding mutant site. **c** Luciferase assays in 293T cells in the indicated conditions. **d** The expression of ARFGEF1 was detected in PTC tissues (T: tumor) and the corresponding normal tissues (A: adjacent normal tissues) by western blotting. **e** Relative expression of ARFGEF1 in Nthy-ori 3–1 and PTC cell lines. **f** IHC staining demonstrated that ARFGEF1 expression was negatively correlated with miR-215 expression (×200). **g** Correlation analysis between miR-215 and ARFGEF1. All experiments were performed in triplicate, and the results are presented as the mean ± SD. **P < 0.01

Western blotting assays indicated that miR-215 inhibition increased the expression of ARFGEF1, while miR-215 overexpression had the opposite result (Fig. 4a). Next, we performed luciferase reporter assays to verify whether *ARFGEF1* is a direct target of miR-215 (Fig. 4b and Supplementary Figure 3). These assays showed that the activity of a luciferase reporter plasmid with the wild-type 3′-UTR of *ARFGEF1* upstream the luciferase coding sequence was significantly suppressed by miR-215 mimics. However, miR-215 mimics did not exert this effect on a luciferase reporter plasmid containing *ARFGEF1*-mutated 3′-UTR (Fig. 4c).

We also detected the expression of ARFGEF1 in human PTC tissues and cell lines by western blotting and found that ARFGEF1 was upregulated in PTC tissues and cells, as compared with the ANT and Nthy-ori 3–1, respectively (Fig. 4d, e). In addition, IHC staining indicated that ARFGEF1 expression was obviously higher in PTC tissues with low miR-215 expression than in those with high miR-215 expression (Fig. 4f). Correlation analysis revealed a negative correlation between miR-215 and ARFGEF1 (r = −0.3219, *P* < 0.001; Fig. 4g). Taken together, these data demonstrated that *ARFGEF1* is a direct downstream target of miR-215 in PTC.

### miR-215 inhibits PTC growth and metastasis by targeting ARFGEF1

**Fig. 5:**
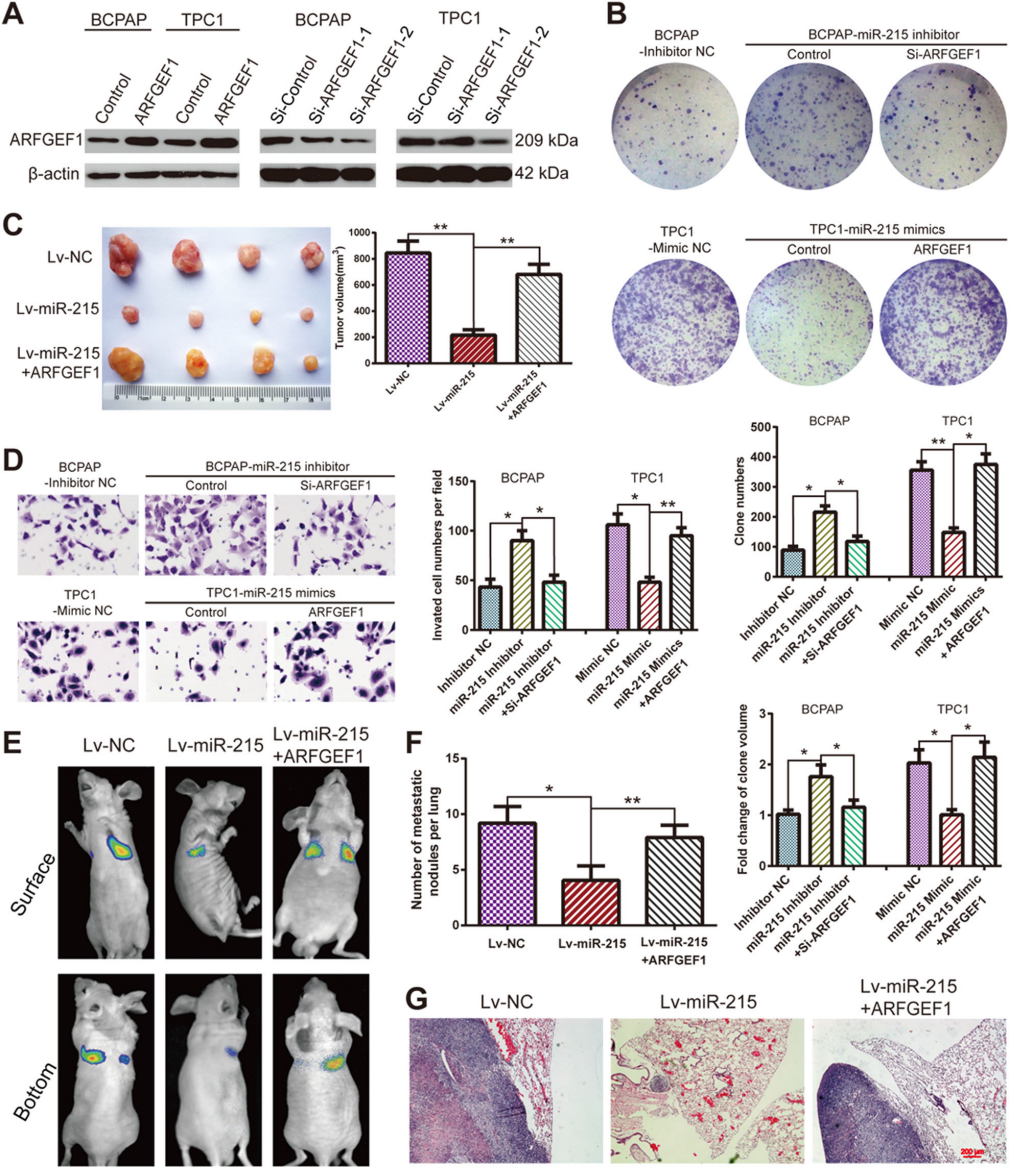
miR-215 exerts its function by modulating ARFGEF1. **a** Western blotting analysis showing the levels of ARFGEF1 after ARFGEF1 overexpression or silencing in PTC cells. **b** Representative images (upper panel) and statistical analysis (lower panel) of colony-formation assays using the indicated PTC cells. **c** Representative photographs and tumor volume of subcutaneous xenografts. **d** The invasive capacities of PTC cells, treated as indicated, were analyzed by transwell invasion assays. **e** Representative bioluminescence images of the lung metastasis model. **f** Number of lung metastatic nodules in the indicated groups (*n* = 6 per group). **g** Representative H&E staining images of lung metastasis samples (×40). All experiments were performed in triplicate, and the results are presented as the mean ± SD. *P < 0.05 and **P < 0.01

To further investigate whether *ARFGEF1* is the functional target of miR-215 in PTC, we overexpressed or silenced ARFGEF1 in BCPAP and TPC-1 cells, and investigated the subsequent effect on proliferation and metastasis. We tested two siRNAs targeting *ARFGEF1*. Western blotting and qPCR assays showed that si-ARFGEF1–2 was more efficient in knocking down ARFGEF1 in PTC cells: this siRNA was therefore chosen for further experiments (Fig. 5a and Supplementary Figure 4). Colony-formation assays revealed that silencing *ARFGEF1* inhibited the proliferation of BCPAP and TPC-1 (Supplementary Figure 5). Colony-formation and CCK-8 assays indicated that the downregulation of *ARFGEF1* counteracts the enhanced proliferation mediated by miR-215 inhibition. On the contrary, upregulation of ARFGEF1 restores the inhibitory effect on proliferation mediated by miR-215 overexpression (Fig. 5b and Supplementary Figure 6). The xenograft tumor model further confirmed these results (Fig. 5c).

Matrigel invasion and transwell migration assays revealed that the increased cell mobility associated with miR-215 inhibition was attenuated by *ARFGEF1* silencing, while ARFGEF1 overexpression counteracted the effects of miR-215 overexpression (Fig. 5d and Supplementary Figure 7). Furthermore, we performed experiments on the nude mice lung metastatic model to confirm these results in vivo. We found that the number of lung metastatic nodules in mice injected with TPC-1 cells was markedly decreased by miR-215 overexpression and significantly increased by ARFGEF1 overexpression (Fig. 5e–g). These data suggested that miR-215 exerts its function via *ARFGEF1* in PTC tumorigenicity and metastasis both in vitro and in vivo.

### miR-215 represses PTC metastasis by modulating EMT via the ARFGEF1/AKT/GSK-3β signaling

**Fig. 6:**
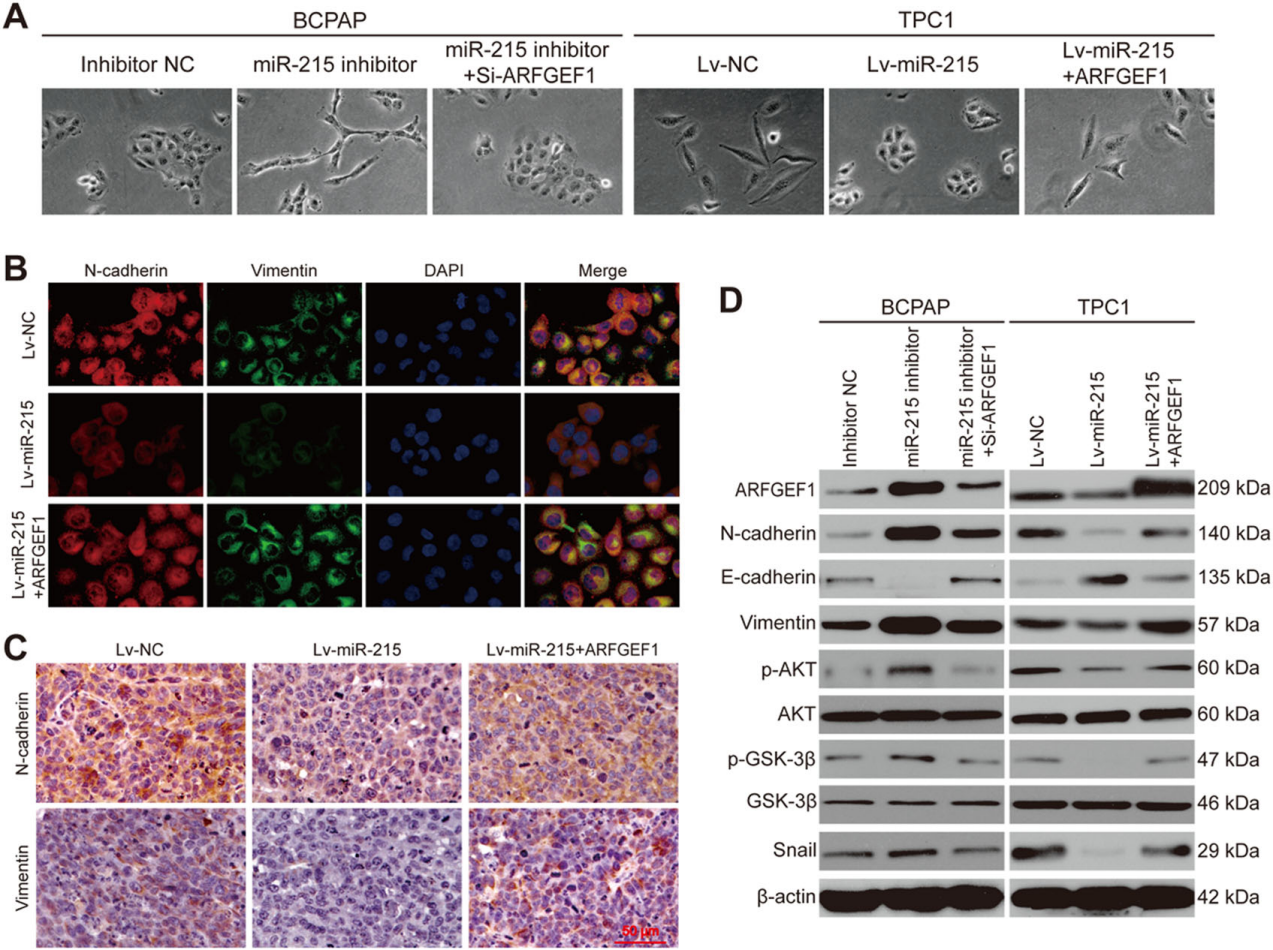
miR-215 inhibits EMT in PTC by modulating the ARFGEF1/AKT/GSK-3β/Snail pathway. **a** Representative morphology images of the indicated PTC cells. **b** Representative IF staining images relative to N-cadherin and vimentin expression in the indicated PTC cells (×200). **c** Representative IHC images relative to N-cadherin and vimentin expression in the indicated tumor sections (×200). **d** Western blotting analysis of protein expression in the indicated PTC cells

EMT plays an important role in tumor metastasis. Therefore, we tested the potential relationship between miR-215 and EMT. Morphology images demonstrated that treatment of BCPAP cells with a miR-215 inhibitor promotes a spindle-like (mesenchymal) morphology, whereas ARFGEF1 silencing restores a round (epithelial) morphology in BCPAP cells. On the other hand, TPC-1-Lv-miR-215 cells appeared rounder than TPC-1-NC and TPC-1-Lv-miR-215-ARFGEF1 cells, overexpressing both miR-215 and ARFGEF1 (Fig. 6a).

Next, we investigated the expression of classic EMT markers by IF and IHC staining in cultured cells and xenograft tumor tissues, respectively. As expected, miR-215 overexpression inhibited the expression of N-cadherin and vimentin, whereas ARFGEF1 overexpression elevated their expression (Fig. 6b, c). These data indicated the negative association between miR-215 and EMT, and were further confirmed by western blotting assays (Fig. 6d).

Since Snail, ZEB1, ZEB2, Slug, and Twist1 were important EMT transcription factors, we took further investigation into the association between miR-215 and these transcriptional regulators. Western blotting analysis revealed that the expression of Snail was elevated or decreased on the condition that miR-215 was downregulated or overexpressed. But no noticeable alteration was found in other transcription factors, including ZEB2, which had been reported to be a direct target of miR-215 in lung cancer and pancreatic cancer^11,12^ (Supplementary Figure 8). In addition, the results from correlation analysis indicated that there was no obvious correlation between miR-215 and ZEB2 in PTC patients (r = 0.05866, *P* > 0.05; Supplementary Figure 9).

It has been reported that ARFGEF1 regulates the AKT signal pathway^13^. Moreover, several studies revealed that the AKT/GSK-3β/Snail signaling was involved in EMT. Therefore, we wondered whether miR-215 could suppress PTC metastasis by modulating EMT via the AKT signaling pathway. To validate that hypothesis, we performed western blotting assays. We found that miR-215 inhibition increases the expression of ARFGEF1, p-AKT, p-GSK-3β, and Snail; in contrast, miR-215 overexpression decreases the levels of ARFGEF1, p-AKT, p-GSK-3β, and Snail (Fig. 6d). However, miR-215 overexpression or inhibition did not affect the levels of the total AKT and GSK-3β. Together, these data indicated that miR-215 inhibits PTC metastasis by repressing EMT via the ARFGEF1/AKT/GSK-3β/Snail signaling (Supplementary Figure 10).

## Discussion

The incidence of PTC is steadily increasing, and recurrence and metastasis hinder its favorable prognosis after treatment^14^. Many miRNAs have been proved to serve as biomarkers for disease diagnosis and prognosis in malignant tumors, including PTC^15,16^. However, the role of miR-215 in PTC remains undefined. In the present study, we show that miR-215 inhibits tumorigenesis and metastasis of PTC through the regulation of the AKT/GSK-3β/Snail signaling by targeting *ARFGEF1*.

Previous studies have shown that the deregulation of miR-215 exerts an important role in some cancers. Georges et al. demonstrated that miR-215 can inhibit colon cancer proliferation by regulating the p53 network^17^. In addition, miR-215 has been reported as a tumor suppressor in osteosarcoma, breast cancer, and intestinal tumor organoids^18–21^. On the contrary, several other studies have described miR-215 as an oncogene-promoting malignant progression in pancreatic carcinomas, gastric cancer, and glioma^22–25^. Here, we proved that miR-215 was significantly downregulated in PTC tissues and cell lines; our data also indicated that miR-215 downregulation is associated with PTC tumor size, metastasis status, and PFS.

We conducted gain- and loss-of-function studies to reveal the role of miR-215 in PTC. We found that miR-215 inhibits proliferation, as validated by CCK-8 and colony-formation assays, and further verified these data in a subcutaneous xenograft model. Meanwhile, miR-215 inhibits the metastasis of PTC, as demonstrated in wound-healing and transwell assays, and in an in vivo lung metastasis model.

It is well recognized that miRNAs exert their function by post transcriptionally modulating their mRNA target^26^. Therefore, we attempted identifying the miR-215 target gene by using publicly available databases. Among the candidates, ARFGEF1 was screened for further studies. We showed that miR-215 inhibits *ARFGEF1* endogenous expression. Dual luciferase assays proved that miR-215 directly targets *ARFGEF1* by complementarily binding to its 3′-UTR. Rescue experiments indicated that *ARFGEF1* knockdown partly mimicked the inhibitory function of miR-215, while ARFGEF1 overexpression attenuated the effects of miR-215 overexpression, demonstrating that ARFGEF1 mediates the activity of miR-215.

EMT plays an important role in tumor metastasis^27^. We checked the expression of classic markers of EMT, and as expected, we found that miR-215 overexpression or *ARFGEF1* knockdown inhibited EMT, whereas, miR-215 silencing and ARFGEF1 ectopic expression promoted it. Several EMT key transcriptional factors, including Snail and ZEB2, displayed a significant role in modulating the EMT process, among which, Snail was detected to be distinctively suppressed by miR-215 in PTC. Despite the fact that ZEB2 was confirmed as a direct target of miR-215 in several other studies^11,12,28^, we found that there was no significant correlation between miR-215 and ZEB2 in PTC.

*ARFGEF1*, also called Brefeldin A-inhibited guanine nucleotide-exchange factor 1 (BIG1), is a high-molecular-weight ADP-ribosylation-factor-specific guanine nucleotide-exchange factor (ARF-GEF)^29,30^. It has been reported that BIG1 plays a critical role in neurite development^13^, initiation of myelination^31^, and membrane trafficking, and contributes to cell adhesion and migration^32–34^. Recently, Matsuyama et al. have revealed that *ARFGEF1* is a direct target of miR-27b, and promotes cell proliferation in colon cancer by activating the Akt pathway^35^.

Meanwhile, the AKT/GSK-3β/Snail axis is important in modulating EMT^36,37^. We found that miR-215 can inhibit AKT phosphorylation mediated by ARFGEF1, and subsequently impair GSK-3β phosphorylation and Snail expression. Thereby, we demonstrated that miR-215 inhibits EMT via the ARFGEF1-activated AKT/GSK-3β/Snail signaling.

In conclusion, we demonstrated that miR-215 was downregulated in PTC tissues and cell lines, and was negatively associated with prognosis in patients with PTC. Moreover, miR-215 inhibited proliferation and metastasis in vitro and in vivo by suppressing *ARFGEF1* and inhibiting the AKT/GSK-3β/Snail signaling pathway. Finally, our research suggests that miR-215 may be a novel prognostic marker for PTC.

## Materials and methods

### Patient samples

Forty-eight pairs of human PTC and ANT were collected from the Department of Head and Neck Surgery, The Third Affiliated Hospital of Harbin Medical University (China) from May 2016 to August 2017. PTC samples were classified based on World Health Organization criteria. Informed consent was obtained by all patients whose biological samples were used in the study, and the experimental protocol for human subjects was approved by the Research Ethics Committee of the Third Affiliated Hospital of Harbin Medical University.

### Quantitative PCR (qPCR)

The total RNA of tissues and cultured cells was extracted with the RNAeasy Mini Kit (Qiagen, Valencia, CA, USA) according to the manufacturer’s instructions, and reverse-transcribed with the TaqMan MicroRNA Reverse Transcription Kit or High Capacity Reverse Transcription Kit (both from Applied Biosystems, Foster City, CA, USA). qPCR assays were performed in duplicate with TaqMan Universal PCR Master Mix II no UNG (Applied Biosystems) or GoTaq qPCR Master Mix (Promega, Madison, WI, USA) on the ABI7900HT Real-Time PCR system (Applied Biosystems). Data were analyzed using the 2^–ΔΔCt^ method. The miRNA and mRNA expression levels were normalized to U6 and glyceraldehyde 3 phosphate dehydrogenase (GAPDH), respectively. The probes for U6 and miR-215 (Cat#: 4426961) were purchased from Thermofisher Scientific (San Jose, CA, USA). The sequence of primers used for qPCR were *ARFGEF1* Fwd: 5′-CGAACATCAAAGTCCACC-3′ and Rev: 5′-ACAGACCACAAGCACCAC-3′; *ZEB2* Fwd: 5′-TTCCTGGGCTACGACCATACC-3′ and Rev: 5′-CAAGCAATTCTCCCTGAAATCC-3′; *GAPDH* Fwd: 5′-GCACCGTCAAGGCTGAGAAC-3′ and Rev: 5′-TGGTGAAGACGCCAGTGGA-3′.

### Cell culture

The human normal thyroid epithelial cell line Nthy-ori 3–1 was purchased from the European Collection of Cell Culture (ECACC, Salisbury, UK). PTC cell lines (TPC-1, K1, BCPAP, and IHH4) were purchased from Cell Bank of Type Culture Collection of the Chinese Academy of Sciences, Shanghai Institute of Cell Biology (Shanghai, China). TPC-1, BCPAP, and IHH4 cells were cultured in Roswell Park Memorial Institute medium (RPMI) 1640 supplemented with 10% fetal bovine serum (FBS), Nthy-ori 3–1 cells were cultured in F12K medium supplemented with 10% FBS, and K1 cells were cultured in Dulbecco’s modified Eagle’s medium (DMEM) supplemented with 10% FBS. All the cells were incubated in a 5% CO_2_ incubator at 37 °C.

### Lentivirus and reagents

miR-215 mimics, miR-215 inhibitor, and their corresponding negative control (NC) oligonucleotide were synthesized by RiboBio Co. Ltd. (Guangzhou, China). Small interfering RNA against *ARFGEF1* (si-ARFGEF1) and nonspecific NC siRNA were purchased from GenePharma Co., Ltd. (Shanghai). Lentiviral vectors for human miR-215 and ARFGEF1 overexpression (Lv-miR-215 and Lv-ARFGEF1) or the plasmids for luciferase reporter study were purchased from GeneChem Co. Ltd. (Shanghai). After the cells were infected with lentiviral vectors, we selected the cells for 2 weeks with 1 μg/mL puromycin (Sigma-Aldrich, St. Louis, MO, USA).

### Cell counting kit-8 (CCK8) and colony-formation assays

Cell proliferation was assessed using the CCK8 kit (Dojindo, Kumamoto, Japan). The cells were seeded in a 96-well plate at a density of 2,000–4,000 per well and their viability was determined 24–72 h later. The cell medium was replaced with 100 μL of complete medium supplemented with 10 μL of CCK8, and the cells were incubated at 37 °C with 5% CO_2_ for 2 h. Absorbance values (OD) were measured at 450 nm in a microplate reader (SpectraMax M2, Molecular Devices, CA, USA).

Colony-formation assays were performed using 0.5% crystal violet, after the cells were plated in six-well plates at a density of 500 cells per well and cultured at 37 °C with 5% CO_2_ for 14 days. Images were captured with a Nikon microscope (Tokyo, Japan).

### Wound-healing assays

Cells were seeded in six-well plates and allowed to grow to confluence. The monolayer was scratched with a micropipette tip, the cells were washed three times in a medium, and images of the wound area were captured at 0 and 24 h with an Eclipse microscope (Nikon).

### Transwell migration and invasion assays

Transwell chambers (Corning, NY, USA) were used to perform transwell migration assays. Cells were seeded in serum-free media into the upper chamber and maintained at 37 °C. The bottom of the chamber was filled with normal media. Matrigel (BD Biosciences, Franklin Lakes, NJ, USA) was used to pre-coat the chamber membrane in 24-well dishes for the invasion assays. The chambers were maintained in a 5% CO_2_ incubator at 37 °C for 24–48 h. The cells on the lower side of the filter were then stained with 0.5% crystal violet and counted under a light microscope (Olympus, Tokyo, Japan, ×200 magnification).

### Western blot analysis

Total proteins from the cells or tissues were extracted using a protein lysis buffer with PhosStop phosphatase inhibitor cocktail and protease inhibitor cocktail (both from Roche Diagnostics, Indianapolis, IN, USA). Lysates were denatured prior to sodium dodecyl sulfate polyacrylamide gel electrophoresis (SDS-PAGE) and then transferred to polyvinylidene difluoride (PVDF) membranes (Millipore, Hertfordshire, UK). The membranes were blocked with 5% nonfat milk at 20–25 °C for 1 h and then incubated with primary antibodies overnight at 4 °C. The following day, the membranes were washed and further incubated with secondary antibodies for 1 h. The immunoreactive signals were visualized using enhanced chemiluminescence reagents (ECL, Pierce, Rockford, USA). The following antibodies were used: anti-ARFGEF1 (Abcam, ab183747, Cambridge, MA, USA), anti-Ki-67 (Abcam, ab15580), anti-Vimentin (Cell Signaling, #3932, Beverly, MA, USA), anti-E-cadherin (Cell Signaling, #3195), anti-N-Cadherin (Cell Signaling, #13116), anti-Snail (Cell Signaling, #3879), anti-Slug (Cell Signaling, #9585), anti-ZEB1 (Cell Signaling, #3396), anti-Twist1 (Cell Signaling, #46702), anti-ZEB2 (Abcam, ab138222), anti-AKT (Cell Signaling, #4685), anti-p-AKT (Cell Signaling, #4060), anti-GSK-3β (Abcam, ab32391), anti-p-GSK-3β (Abcam, ab75745), and anti-β-actin (Sigma, A5441).

### Immunohistochemistry (IHC) assays

Tissues were fixed with 10% formalin and embedded in paraffin. Tissue sections were deparaffinized in xylene, rehydrated in a serial of graded ethanol, and antigens were retrieved by boiling in an antigen unmasking solution (Vector lab, Burlingame, CA, USA). Endogenous peroxidase activity was blocked with 10% goat serum for 30 min, after which the tissue sections were incubated with a primary antibody at 4 °C overnight, and then with a secondary antibody (Vector Lab) for 1 h. The immunocomplexes were visualized with a 3,3′-diaminobenzidine tetrahydrochloride solution (DAB kit, Vector lab) and the sections were stained with hematoxylin (Sigma). Slides were dehydrated through ethanol, sealed with coverslips, and imaged under a light microscope (Olympus, ×200 magnification).

### Luciferase reporter assays

Cells were seeded in 24-well plates and co-transfected with the plasmids containing the wild-type or mutated 3′-UTR sequence of *ARFGEF1*, pRL-TK Renilla, and miR-215 mimics using Lipofectamine 3000 (Invitrogen). The luciferase activity was measured after incubation for 48 h with the Dual Luciferase Reporter Assay System (Promega) according to the manufacturer’s instructions.

### Immunofluorescence (IF) assays

PTC cells were fixed with 4% paraformaldehyde, permeabilized with 0.5% Triton X-100 (Sigma), blocked by 10% normal goat serum (Vector Lab), and incubated with primary antibodies overnight at 4 °C. Then, the cells were incubated with Alexa Fluor 488- or 555-conjugated secondary antibodies (Invitrogen), stained with DAPI (Vector Lab), and photographed under a fluorescence microscope (Olympus).

### Xenograft nude mouse model

All-male BALB/c athymic nude mice (4–6 weeks old) were purchased from the Experimental Animal Center of Shanghai Institute for Biological Sciences (China) and maintained in pathogen-free conditions. The procedures were performed in accordance with the institutional guidelines for animal care, and approved by the Committee on the Use of Live Animals in Teaching and Research of the Harbin Medical University.

To establish the subcutaneous xenograft model, ~5 × 10^6^ tumor cells in 0.1 mL of phosphate-buffered saline were injected subcutaneously into the flanks of the nude mice. Four weeks later, the animals were euthanized, and the tumors were excised and weighed. The tumor volume was calculated using the equation: volume = width^2^ × length × 0.5 (mm^3^).

For the in vivo metastasis assays, 5 × 10^6^ cells were injected into the lateral tail vein of nude mice. The mice were imaged after 6 weeks using a NightOWL LB983 bioluminescence system (Berthold Technologies, Wildbad, Germany) upon intraperitoneal injection of D‑luciferin (Promega) under anesthesia. Eight weeks later, the mice were killed, and the lungs were resected and fixed in 10% formalin for hematoxylin and eosin (H&E) staining.

### Statistical analysis

The results are presented as mean values ± standard deviation (SD) of at least three independent experiments and were analyzed with SPSS 20.0 software (SPSS, Chicago, IL, USA) or Prism 6.0 software (GraphPad, San Diego, CA, USA). Statistical significance between two groups was analyzed using the Student’s *t* test, while multiple-group comparison was performed with one-way analysis of variance (ANOVA). *P-*values less than 0.05 were considered statistically significant.

## Supplementary information

Supplementary Information

Supplementary Table 1

Supplementary Figure 1

Supplementary Figure 2

Supplementary Figure 3

Supplementary Figure 4

Supplementary Figure 5

Supplementary Figure 6

Supplementary Figure 7

Supplementary Figure 8

Supplementary Figure 9

Supplementary Figure 10

Supplemental figure legends
