# Supplementary material for: miR-215 suppresses papillary thyroid cancer proliferation, migration, and invasion through the AKT/GSK-3β/Snail signaling by targeting ARFGEF1
Source: Cell Death Dis. 2019 Feb 27;10(3):195. doi: 10.1038/s41419-019-1444-1 (PMC6393497; doi:10.1038/s41419-019-1444-1)
Supplement: Supplementary file 1 — Supplementary Information [file 41419_2019_1444_MOESM1_ESM.docx]

**Supplementary Information**

**Supplementary Table 1 Correlation between miR-215 and the clinicopathologic characteristics of 48 patients with PTC**

**Supplementary Figure 1** **Colony formation of indicated PTC cells, after different concentration of miR-215 mimics oligonucleotides transfection**. Representative images (left) and statistical analysis (right) of colony formation from the indicated PTC cells. All experiments were performed in triplicate, and the results are presented as the mean ± SD. **P* < 0.05, and ***P* < 0.01

**Supplementary Figure 2** **The migration capacities of PTC cells, treated as indicated, were detected by transwell assays**. All experiments were performed in triplicate, and the results are presented as the mean ± SD. **P* < 0.05, ***P* < 0.01, and ****P* < 0.001

**Supplementary Figure 3 The entire recognition site of *ARFGEF1*, the** **highlighted sequences are the seeds of miR-215**

**Supplementary Figure 4** **Relative expression of *ARFGEF1* after ARFGEF1 overexpression or silencing in PTC cells, as measured by qPCR**. All experiments were performed in triplicate, and the results are presented as the mean ± SD. **P* < 0.05, ***P* < 0.01, and ****P* < 0.001

**Supplementary Figure 5 Representative images and statistical analysis of colony formation assays using the indicated PTC cells.** All experiments were performed in triplicate, and the results are presented as the mean ± SD. **P* < 0.05, and ***P* < 0.01

**Supplementary Figure 6** **Growth curves obtained in proliferation (CCK8) assays in the indicated PTC cells**

**Supplementary Figure 7** **The migration capacities of PTC cells, treated as indicated, were detected by transwell assays**. All experiments were performed in triplicate, and the results are presented as the mean ± SD. **P* < 0.05, and ***P* < 0.01

**Supplementary Figure 8 Western blotting analysis of EMT transcription factors protein expression in the indicated PTC cells**

**Supplementary Figure 9 Correlation analysis between miR-215 and ZEB2**

**Supplementary Figure 10 Schematic presentation of mechanism underlying miR-215-mediated PTC metastasis**
