# Supplementary material for: miR-215 suppresses papillary thyroid cancer proliferation, migration, and invasion through the AKT/GSK-3β/Snail signaling by targeting ARFGEF1
Source: Cell Death Dis. 2019 Feb 27;10(3):195. doi: 10.1038/s41419-019-1444-1 (PMC6393497; doi:10.1038/s41419-019-1444-1)
Supplement: Supplementary file 2 — Supplementary Table 1 [file 41419_2019_1444_MOESM2_ESM.docx]

**Supplementary Table 1 Correlation between miR-215 and the clinicopathologic characteristics of 48 patients with PTC.**

| Features | Relative miR-215 expression | | *P* Value |
| --- | --- | --- | --- |
|  | Low  (n = 33) | High  (n = 15) |  |
| Age (years) |  |  | > 0.05 |
| ≤ 45 | 13 | 6 |  |
| > 45 | 20 | 9 |  |
| Gender |  |  | > 0.05 |
| Male | 8 | 5 |  |
| Female | 25 | 10 |  |
| Extrathyroidal extension |  |  | > 0.05 |
| Yes | 10 | 4 |  |
| No | 23 | 11 |  |
| Tumor size (cm) |  |  | 0.0137 |
| ≤ 1 | 11 | 11 |  |
| > 1 | 22 | 4 |  |
| Differentiation |  |  | 0.0274 |
| I~II | 10 | 10 |  |
| III~IV | 23 | 5 |  |
| Lymph node metastasis |  |  | 0.0032 |
| Yes | 25 | 4 |  |
| No | 8 | 11 |  |

Significance figures are *P* values for Fisher’s exact test.
