# Supplementary figures and images for: miR-215 suppresses papillary thyroid cancer proliferation, migration, and invasion through the AKT/GSK-3β/Snail signaling by targeting ARFGEF1
Source: Cell Death Dis. 2019 Feb 27;10(3):195. doi: 10.1038/s41419-019-1444-1 (PMC6393497; doi:10.1038/s41419-019-1444-1)

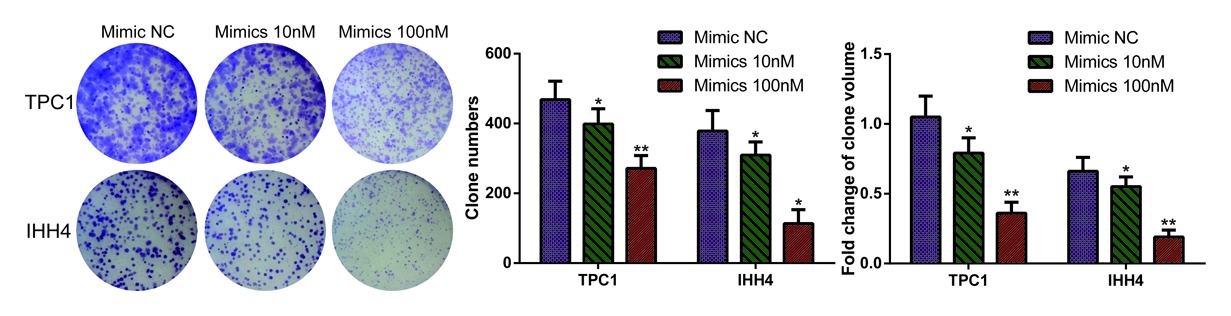

Supplement: Supplementary file 3 — Supplementary Figure 1 [file 41419_2019_1444_MOESM3_ESM.tif]

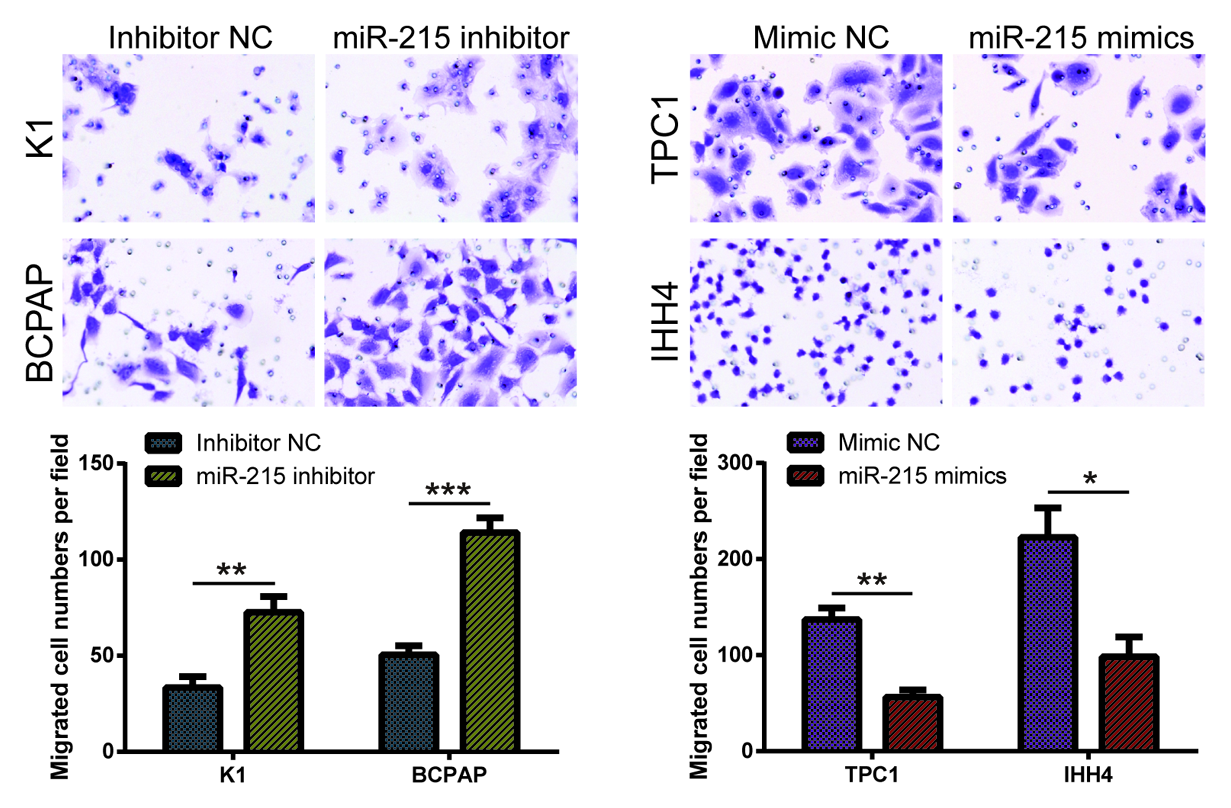

Supplement: Supplementary file 4 — Supplementary Figure 2 [file 41419_2019_1444_MOESM4_ESM.tif]

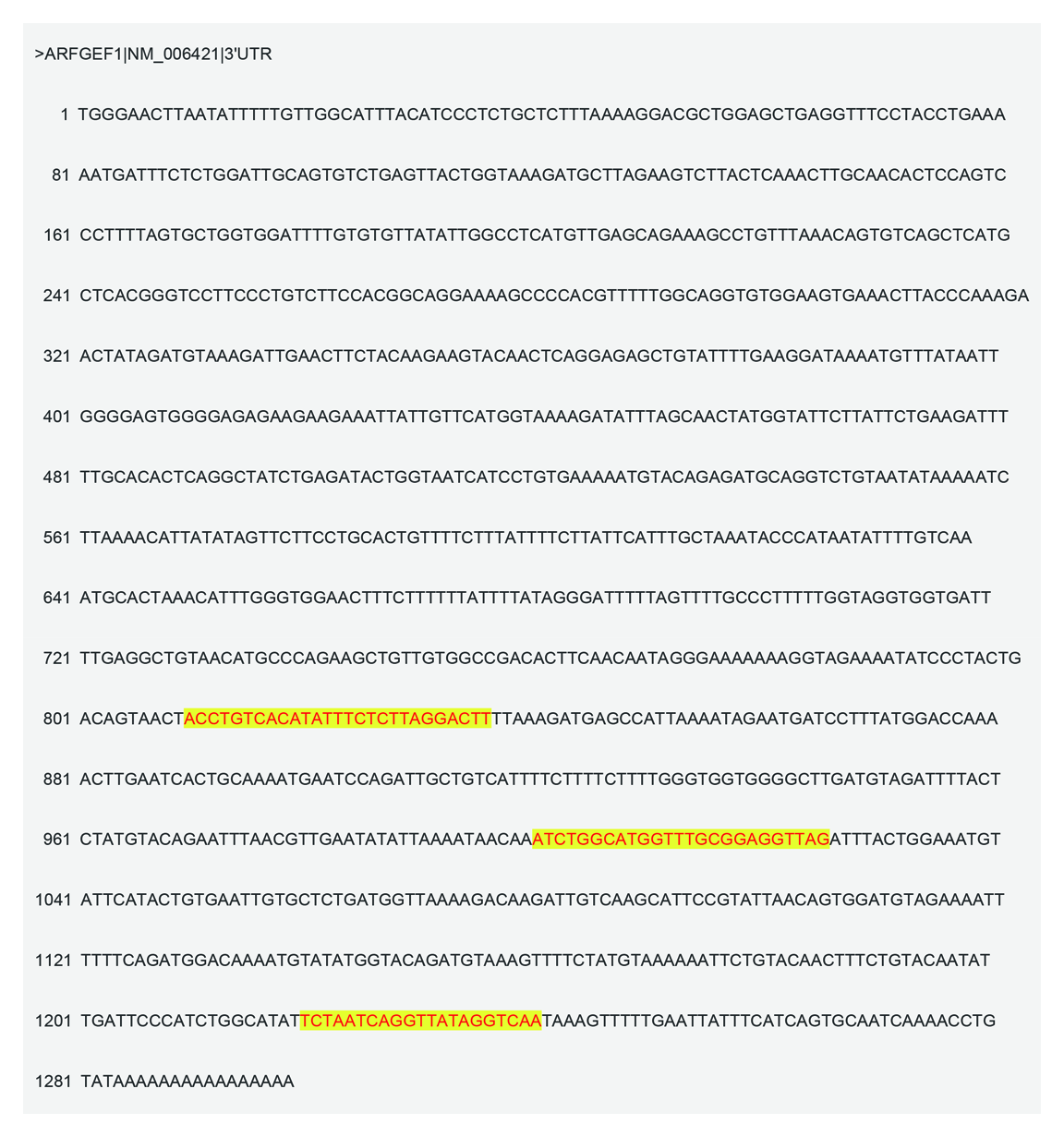

Supplement: Supplementary file 5 — Supplementary Figure 3 [file 41419_2019_1444_MOESM5_ESM.tif]

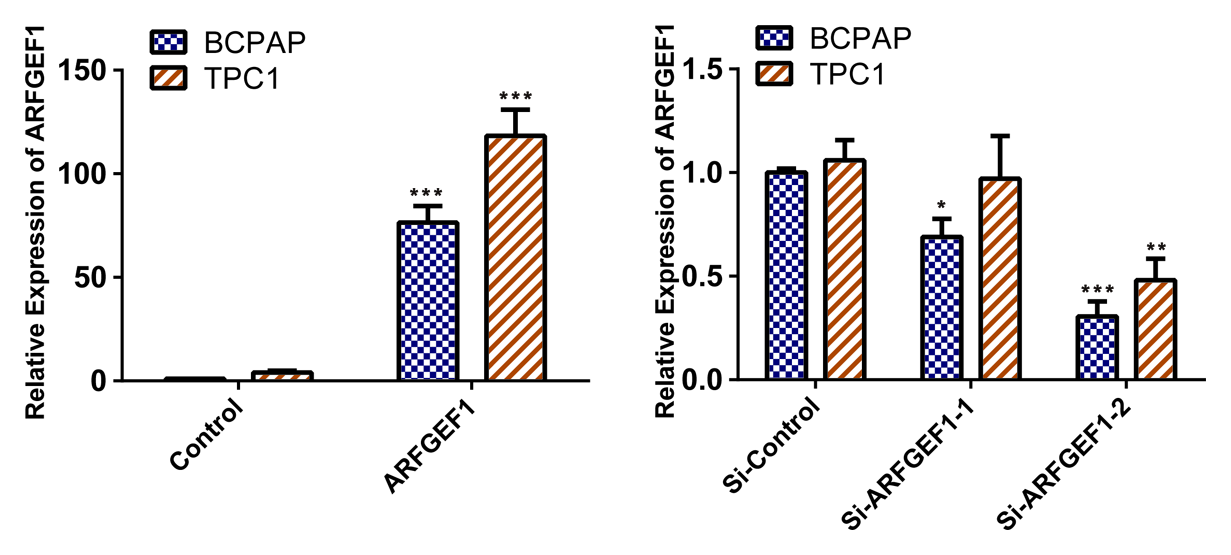

Supplement: Supplementary file 6 — Supplementary Figure 4 [file 41419_2019_1444_MOESM6_ESM.tif]

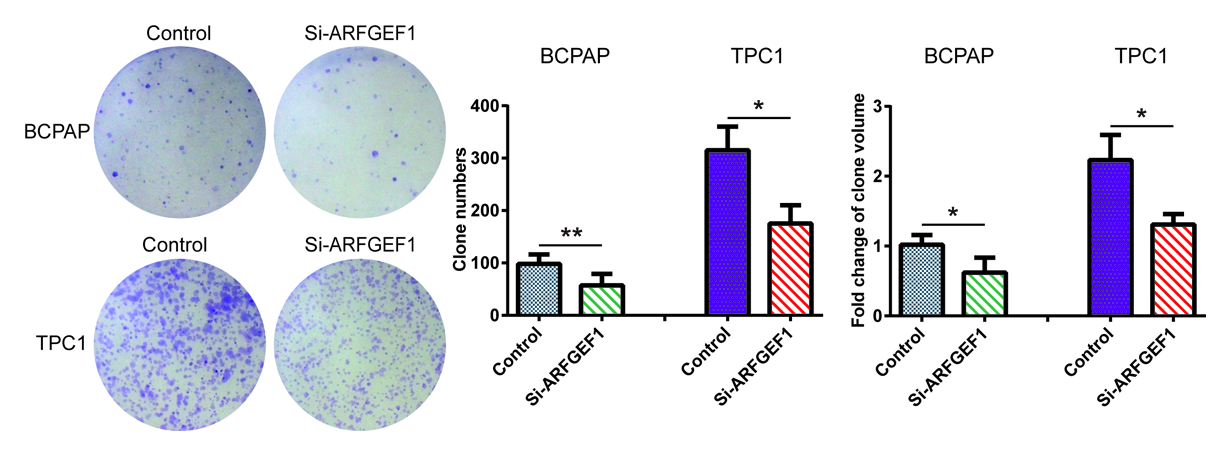

Supplement: Supplementary file 7 — Supplementary Figure 5 [file 41419_2019_1444_MOESM7_ESM.tif]

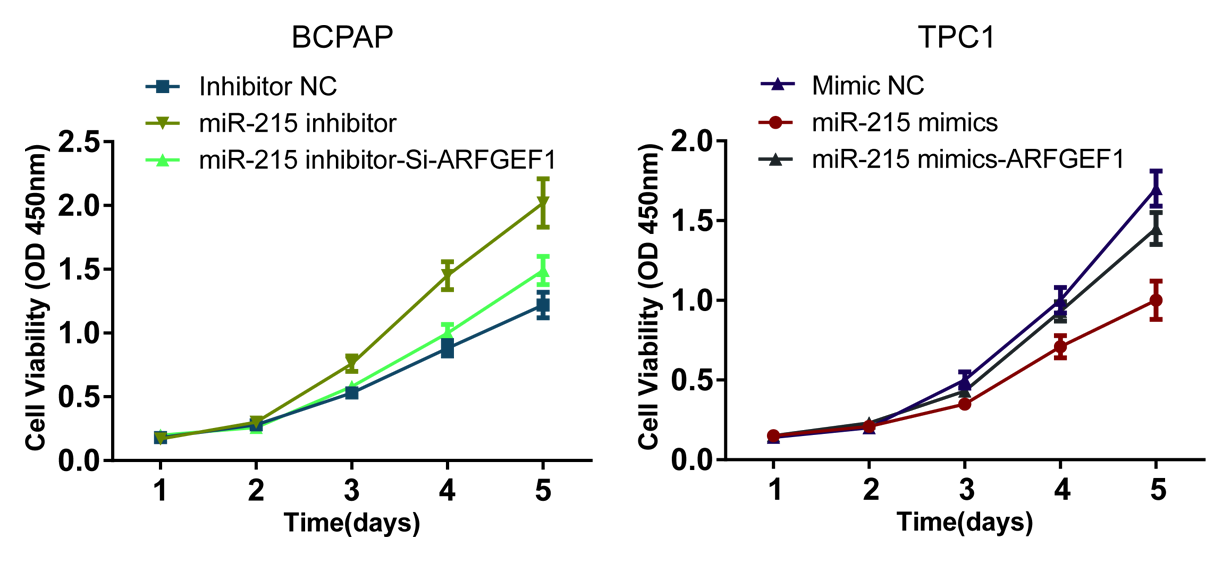

Supplement: Supplementary file 8 — Supplementary Figure 6 [file 41419_2019_1444_MOESM8_ESM.tif]

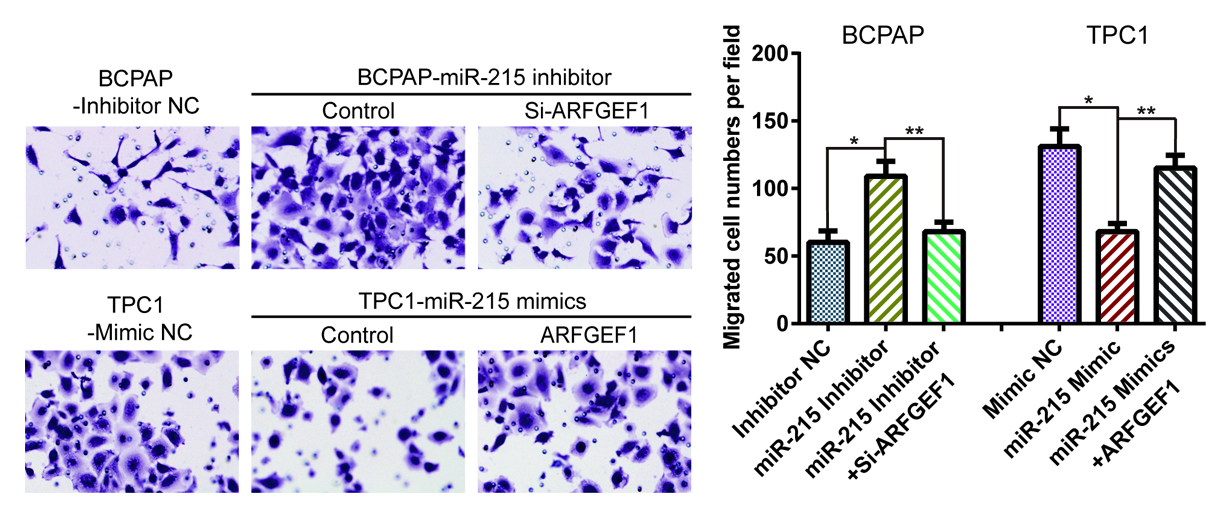

Supplement: Supplementary file 9 — Supplementary Figure 7 [file 41419_2019_1444_MOESM9_ESM.tif]

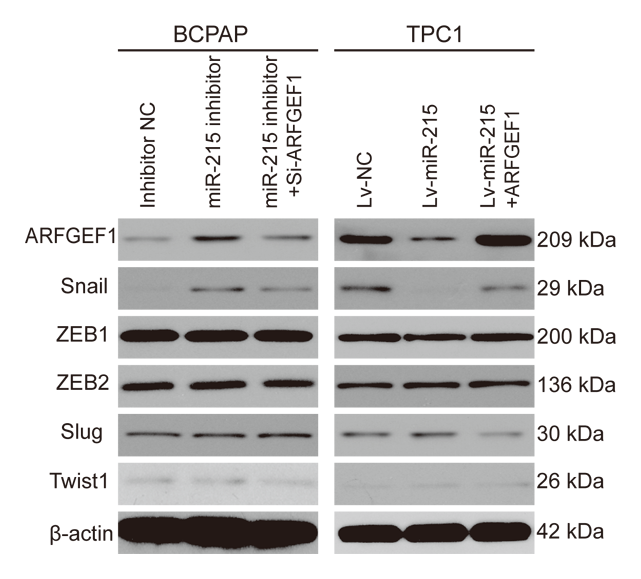

Supplement: Supplementary file 10 — Supplementary Figure 8 [file 41419_2019_1444_MOESM10_ESM.tif]

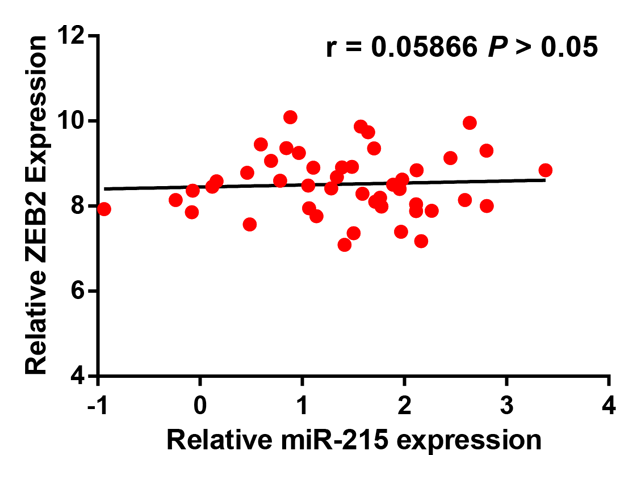

Supplement: Supplementary file 11 — Supplementary Figure 9 [file 41419_2019_1444_MOESM11_ESM.tif]

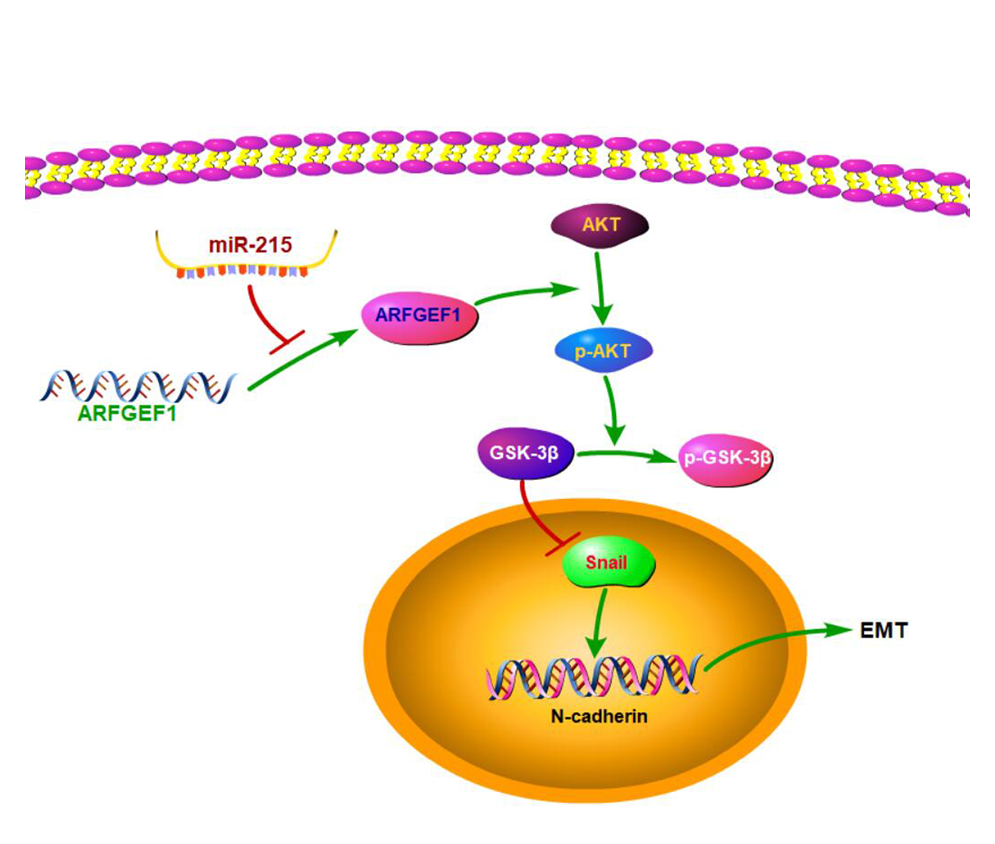

Supplement: Supplementary file 12 — Supplementary Figure 10 [file 41419_2019_1444_MOESM12_ESM.tif]
